# No Significant Differences between Bisphosphonates and Placebo for the Treatment of Bone Marrow Lesions of the Knee: A Systematic Review and Meta-Analysis of Randomized Controlled Trials

**DOI:** 10.3390/jcm13133799

**Published:** 2024-06-28

**Authors:** Giuseppe Anzillotti, Felix C. Öttl, Carlotta Franceschi, Pietro Conte, Enrico Maria Bertolino, Marina Lipina, Alexey Lychagin, Elizaveta Kon, Berardo Di Matteo

**Affiliations:** 1 IRCCS Humanitas Research Hospital, Via Manzoni 56, Rozzano, 20089 Milan, Italy; pietro.conte@humanitas.it (P.C.); elizaveta.kon@humanitas.it (E.K.); berardo.dimatteo@gmail.com (B.D.M.); 2 Department of Biomedical Sciences, Humanitas University, Via Rita Levi Montalcini 4, Pieve Emanuele, 20072 Milan, Italy; carlotta.franceschi@st.hunimed.eu (C.F.); enricobertolino99@gmail.com (E.M.B.); 3 Department of Hip and Knee Surgery, Schulthess Klinik, 8008 Zurich, Switzerland; oettlf@hss.edu; 4 Hospital for Special Surgery, New York, NY 10021, USA; 5 Department of Traumatology, Orthopaedics and Disaster Surgery, Sechenov University, Moscow 119991, Russia; marina.lipina@icloud.com (M.L.); dr.lychagin@mail.ru (A.L.); 6 Laboratory of Clinical Smart Nanotechnologies, Sechenov University, Moscow 119991, Russia

**Keywords:** bisphosphonates, bone marrow lesions, knee, bone marrow edema

## Abstract

**Objectives**: The purpose of the present systematic review and meta-analysis is to summarize the current evidence on the role of bisphosphonates in the treatment of knee bone marrow lesions (BMLs), to understand whether they are truly effective in improving symptoms and restoring the subchondral bone status at imaging evaluation. **Methods**: A literature search was carried out on PubMed, Cochrane, and Google Scholar databases in accordance with the PRISMA guidelines. Potential risk of bias was evaluated using the Cochrane Risk of Bias 2 tool for randomized controlled trials (RCTs) and the ROBINS-I tool for non-randomized studies. **Results**: A total of 15 studies were included in the present systematic review and meta-analysis. Seven studies were RCTs, two were prospective cohort studies, three were retrospective, and three were case series. Our meta-analysis revealed that bisphosphonates did not significantly improve clinical scores or reduce BML size compared to placebo. Accordingly, the rate of adverse events was also non-significantly higher among bisphosphonate users versus placebo users. **Conclusions**: The main finding of the present meta-analysis and systematic review is that bisphosphonates show neither significant benefits nor significant adverse events when compared to placebo in the treatment of BMLs of the knee. **Level of Evidence**: Level IV systematic review of level II–III–IV studies. Level I meta-analysis of level I studies.

## 1. Introduction

Knee bone marrow lesion (BML) is a descriptive term that identifies a magnetic resonance imaging (MRI) finding often associated with severe pain and functional impairment, whose diagnosis is often delayed [1,2,3,4]. Even though the exact pathogenetic processes remain unknown, BMLs represent a core component of various pathological conditions of the knee, e.g., trauma, osteoarthritis (OA) [5], inflammatory arthritis, osteonecrosis, osteochondritis dissecans, neoplasms, and infections [6].

The clinical relevance of BMLs lies in the fact that these lesions are strongly associated with symptom onset and progression, and their persisting presence negatively affects the patient’s prognosis in many diseases, above all knee OA [7,8]. Hence, it has been hypothesized that initiating specific treatments targeting BMLs could improve a patient’s pain and, therefore, activity levels, in an attempt to delay or even reverse further progression of the disease [9].

In terms of diagnostic features, BMLs can be detected at MRI using fluid-sensitive sequences, e.g., fat-suppressed T2-weighted, proton density-weighted, or intermediate-weighted fast spin echo or short tau inversion recovery (STIR) sequences. The most representative imaging findings of BMLs are a high signal intensity within the bone marrow region on fat-suppressed T2-weighted images and a low signal intensity on T1-weighted images [10,11,12].

In selected cases, BMLs may resolve spontaneously over a period of 6–12 months; however most patients tend to experience intense or prolonged pain, thus requiring treatment [13,14,15]. Currently, there is no established treatment algorithm for BMLs, and the available options mainly address symptoms, including restricted weight bearing, pharmacotherapy, extra-corporeal shock-wave therapy, and, eventually, surgery [6,16]. Pharmacotherapy is based on anti-inflammatory and analgesic use dictated by the patient’s pain and, more recently, intravenous prostacyclin or bisphosphonates [6,13].

Bisphosphonates are a class of anti-resorptive medications used in the treatment of osteoporosis that inhibit bone resorption and, consequently, bone remodeling by inducing apoptosis in osteoclasts and preventing apoptosis in osteocytes and osteoblasts [17,18]. In the past two decades, bisphosphonates have gained significant attention as a potential therapeutic intervention in the treatment of knee BMLs to achieve both clinical improvement and prevent disease progression [19,20,21]. Indeed, given their positive effects on bone metabolism, their use for knee BMLs has shown a reduction in lesion size with normalization of MRI, suggesting that these drugs might have a disease-modifying effect [22,23].

The purpose of the present systematic review and meta-analysis is to summarize the current evidence on the role of bisphosphonates in the treatment of knee BMLs, to understand whether they are truly effective in improving symptoms and restoring the subchondral bone status at imaging evaluation.

## 2. Materials and Methods

A literature review was performed following the Preferred Reporting Items for Systematic Reviews and Meta-Analyses (PRISMA) guidelines [24]. The literature search was conducted on PubMed, Embase, and Google Scholar databases up to 16 February 2024, by using the following search string: “bisphosphonates” OR “alendronate” OR “ibandronate” OR “zoledronic acid” OR “zoledronate” AND “bone marrow edema” OR “bone marrow oedema” OR “bone marrow lesion” OR “algodystrophy” OR “transient osteoporosis” OR “BME” OR “BML” OR “osteonecrosis”. Two reviewers (GA and FÖ) independently carried out the screening process. Initially, the articles underwent title and abstract screening, considering the following inclusion criteria: (1) randomized controlled trials (RCTs); retrospective or prospective studies on humans; (2) written in English language; and (3) analyzing the effect of bisphosphonates in the management of BMLs of the knee. Exclusion criteria were articles written in other languages, in vitro studies, animal studies, reviews, and studies on the use of bisphosphonates for BMLs not located in the knee. 

**Figure 1:**
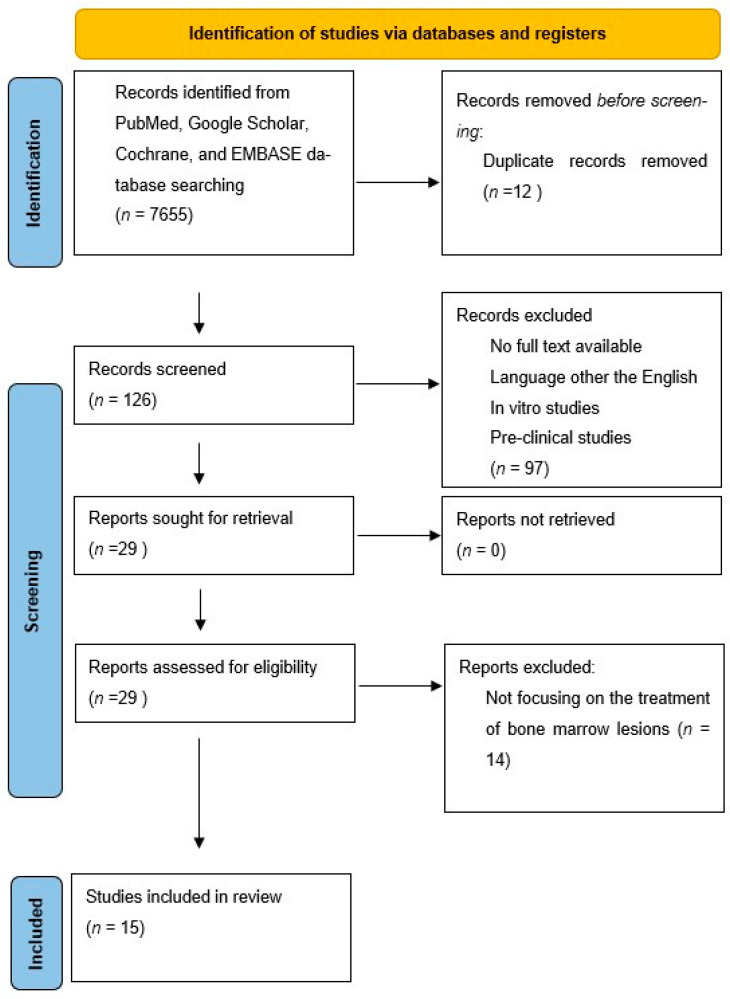
Preferred Reporting Items for Systematic Review and Meta-Analysis (PRISMA) flowchart of the systematic literature review.

Covidence systematic review software (Veritas Health Innovation, Melbourne, Australia) was utilized to screen citations and full-text articles, resolve conflicts, and extract data using customizable forms. Concluding the initial screening phase, full texts of included articles were evaluated and the reference list of all the retrieved articles was further screened for identification of potentially relevant studies. A PRISMA flowchart of the selection process is provided in Figure 1.

Senior investigators were responsible for consensus when discrepancies between the two reviewers were encountered. Included full texts underwent data extraction and subsequent collection, for the purposes of the present manuscript.

The quality of RCTs was assessed through the Cochrane Risk of Bias tool for Randomized Controlled Trials (ROB-2) [25]. Conversely, the risk of bias for prospective and retrospective studies was performed according to the ROBINS-I tool for non-randomized studies [26].

For the meta-analysis, the statistical methodology employed was the inverse variance method, with the effect measure represented as the standardized mean difference. A random effects analysis model was applied to assess the overall effect. No subgroups or filters were implemented in this analysis. For adverse events analysis, we examined the occurrence of any complications, presented in a dichotomous format. The Mantel–Haenszel method was adopted, employing the odds ratio as the effect measure. We then implemented a random effects analysis model to gauge the overall impact.

## 3. Results

Fifteen studies that met the inclusion criteria were included in the present review (Table 1). The included studies examined the effects of bisphosphonates on BMLs and pain in various conditions such as OA, bone marrow edema syndrome, complex regional pain syndrome, and osteonecrosis. Study designs were as follows: seven RCTs, two prospective cohort studies, three retrospective cohort studies, and three case series. Sample sizes ranged from 12 to 290 participants. The bisphosphonates evaluated were ibandronate, zoledronic acid (ZA), neridronate, and alendronate. 

### 3.1. Risk of Bias Assessment

**Figure 2:**
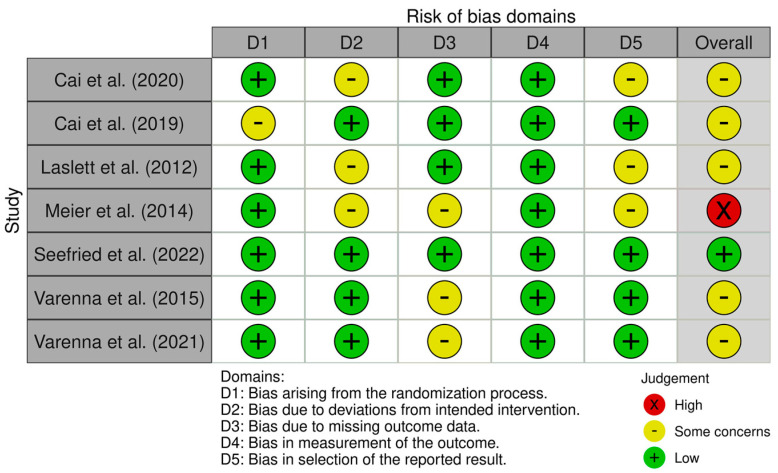
RoB2 tool for the risk of bias assessment of the included RCTs.

The seven different types of bias analyzed in ROB-2 were classified into “low risk”, “high risk”, or “unclear risk”. Afterwards, the results of this assessment were converted to Agency for Healthcare Research and Quality standards, which ultimately rank the RCTs as “good quality”, “fair quality”, and “poor quality”. Based on this, one study was categorized as good quality, five as fair quality, and one as poor quality. The ROBINS-I tool assesses seven different types of bias across seven domains: confounding, selection of participants, classification of interventions, deviations from intended interventions, missing data, measurement of outcomes, and selection of reported results. For each domain, the risk of bias is classified as “low risk”, “moderate risk”, “serious risk”, or “critical risk” based on signaling questions. Afterwards, these domain-level risk judgments are synthesized into an overall risk of bias judgment for the study. The key criteria are: “low risk of bias” if all domains are low risk, “moderate risk of bias” if at least one domain is moderate risk, “serious risk of bias” if at least one domain is serious risk but none are critical risk, and “critical risk of bias” if any domain is critical risk. This ultimately categorizes the non-randomized interventional studies into four tiers of quality: “low risk of bias”, “moderate risk of bias”, “serious risk of bias”, and “critical risk of bias”. Among the studies assessed, one study demonstrated a low risk of bias, four studies had a moderate risk of bias, and one study was judged to have a serious risk of bias (Figure 2 and Table 2).

**Table 1:** List and main characteristics of the included studies.

Publication	Study Design	Level of Evidence	Disease	Therapeutic Protocol	Outcome	Patient Characteristics	Follow-Up	Main Findings
Agarwala et al., 2020 [27]	Prospective case series	IV	Spontaneous osteonecrosis of the knee (SPONK)	Single IV dose of 5 mg of ZA, 70 mg alendronate tablets weekly divided into two doses taken on empty stomach, supplemented with calcium, vitamin D, and anti-inflammatory medications	VAS, Bone marrow involvement (%)	*n* = 16 patients with SPONK lesions involving only the femur	16 weeks	ZA combined with alendronate for SPONK provides faster recovery clinically and radiographically compared to ZA and/or ibandronate alone in the literature
Baier et al., 2013 [1]	Retrospective cohort study	III	BML of the knee, talus, or navicular bone	Ibandronate administered once monthly in a dosage of 6 mg for three consecutive months; iloprost administered intravenously for five consecutive days with 20, 30, or 40 μg	VAS, WOMAC, SF-36	*n* = 20 10 ibandronate, mean age 34.6; 10 iloprost, mean age 35.2	Mean 12 months (range: 10–17)	Conservative treatment of BML in the knee and foot with intravenous infusion of prostacyclin or bisphosphonates represents an encouraging and efficacious option. These infusions are minimally invasive procedures with relatively low risks. Compared to bisphosphonates, prostacyclin appears to act more rapidly and effectively in reducing BML.
Ballal et al., 2020 [28]	Retrospective cohort study	III	OA	Oral bisphosphonate; non-bisphosphonate	BML size (mm^2^)	*n* = 290, propensity score matched; 145 bisphosphonate, age 64.8 (±8.0), BMI 26.3 (±4.3), baseline BML 392.2 mm^2^ (±738.8); 145 non-bisphosphonate, age 65.1 (±7.8), BMI 26.2 (±4.1), baseline BML 355.6 (±704.6)	12 months	The use of bisphosphonates for over 12 months showed no definite harm as a disease-modifying therapy in OA. However, there is insufficient evidence to demonstrate a clear benefit. A potential sign of efficacy was noted among OA patients with BMLs at baseline.
Bartl et al., 2012 [29]	Prospective cohort study	II	BML around knee and ankle joint	Three ambulatory infusions with 6 mg ibandronate; diclofenac sodium, 2 × 75 mg per day for 3 weeks	VAS, Mazur ankle scores, Larson knee scores, BML-stage	*n* = 50 30 ibandronate, 15 knee joint BML, 15 ankle joint BML, mean age 41; 20 diclofenac sodium, mean age 44	12 months	Intravenous ibandronate demonstrated efficacy as a treatment for BMLs of the knee and ankle. Patients receiving ibandronate showed significantly greater improvements in pain and function compared to the control group receiving only analgesic treatment. Follow-up MRI scans revealed a significant decrease in BML size solely in the ibandronate treatment group.
Cai et al., 2020 [30]	Multi-center, double-blind, placebo-controlled RCT	I	>50 years, knee pain, criteria for symptomatic knee OA, subchondral BML present on MRI	Single 15 min intravenous infusion of ZA (5 mg in 100 mL saline solution), identical placebo (100 mL saline solution) at baseline and at 12 months	Tibiofemoral cartilage volume, VAS, WOMAC, BML (mm^2^)	*n* = 223 113 ZA, mean age 62.8 (±8.5), BMI 30.2 (±5.5), BML size 476 mm^2^ (255–860); 110 placebo, mean age 61.3 (±7.3), BMI 30.8 (± 6.2), BML size 502 mm^2^ (225–919)	24 months	Annual ZA infusions did not significantly reduce cartilage volume loss compared to placebo over 24 months.
Cai et al., 2019 [31]	Single-center, double-blind RCT	I	>50 years, knee pain, criteria for symptomatic knee OA, subchondral BML present on MRI	Single infusion of either 5 mg/100 mL ZA/saline for the ZA group and Zobone 5 for the VOLT01 group, or 100 mL saline for the placebo group	Acute phase responses over 3 days, VAS, WOMAC, BML size (mm^2^)	*n* = 117 39 ZA, mean age 64.4 (±8.4), BMI 31 (± 5.2), BML size 446 mm^2^ (±396.7); 40 VOLT01, mean age 60.9 (±8.1), BMI 30.4 (±6), BML size 576.8 mm^2^ (±531.3); 38 placebo, mean age 61.5 (±7.4), BMI 31 (±5.4), BML size 518.8 mm^2^ (±438)	6 months	Intravenous VOLT01 did not reduce APR or knee BML size over 6 months. Unlike ZA alone, this combination may improve symptoms in knee OA.
Laslett et al., 2012 [32]	Single-center, double-blind RCT	I	≥50 years, knee pain, OA, BML on MRI	Intravenous infusion of 100 mg of fluid, containing ZA (5 mg in normal saline) or placebo (normal saline)	VAS, BML size (mm^2^), KOOS, adverse events	*n* = 59 31 ZA, mean age 54.2 (±8.2), BMI 29.6 (±4.4), BML size 483.9 mm^2^ (±410.2); 28 placebo, mean age 60.4 (±7.3), BMI 29.8 (±5.8), BML size 449.4 mm^2^ (±339.3)	12 months	A single ZA infusion reduced knee pain, BML size, and the proportion of patients with clinically significant BML reduction at 6 months.
Meier et al., 2014 [33]	Double-blind RCT	I	Newly diagnosed spontaneous or postarthroscopic osteonecrosis of the knee	13.5 mg ibandronate, placebo intravenously divided in four injections within 2 weeks (once 1.5 mg then 3 mg per injection) and followed by a fifth injection after 3 months (3 mg). In addition, all patients received daily calcium (500 mg) and vitamin D (400 IU) throughout the study and diclofenac (50 mg/day) for the first 3 months.	VAS, WOMAC, IKDC, ALP, PINP, CTX	*n* = 30 14 ibandronate, mean age 62.4 (±7.7), BMI 27.7 (±3.7); 16 placebo, mean age 52.7 (±8.2), BMI 31.1 (±6.2)	48 weeks	Intravenous ibandronate displayed no additional benefit compared to anti-inflammatory medication alone for patients with spontaneous osteonecrosis of the knee.
Müller et al., 2020 [34]	Retrospective cohort study	III	Symptomatic BML of the knee	Vitamin D supplementation, ibandronic acid, ZA, sequential (I→Z), denosumab, alendronic acid	Adverse events, CTX, P1NP, WORMS, “Satisfaction”	*n* = 34 9 ibandronic acid, 12 ZA, 7 sequential (I→Z), 3 denosumab, 3 alendronic acid	4 weeks clinical and laboratory follow-up for all patients, additional follow-up if persistent pain	ZA appeared to exhibit enhanced efficacy compared to alternative antiresorptive medications, particularly ibandronic acid; however, these differences did not achieve statistical significance. Moreover, ZA was associated with a higher frequency of adverse events
Ringe et al., 2005 [35]	Case Series	IV	Localized transient osteoporosis (LTO)	Single IV administration of 4 mg ibandronate; second IV injection of 2 mg ibandronate optional after 3 months, daily calcium (1 g) and vitamin D (800 IU) supplements	VAS, BMD, adverse events	*n* = 12 mean age 44 (range: 29–61),	6 months	IV ibandronate appears effective in treating LTO. It increases BMD locally and systemically while providing significant and substantial pain relief, even in those with prior opioid-resistant pain
Seefried et al., 2022 [36]	Triple-blind, placebo-controlled randomized trial	I	Painful bone marrow lesions	Single-dose ZA 5 mg IV versus placebo, daily calcium 1 g and 1000 IU vitamin D	VAS, BML size, PDI, QoL, biochemical analysis	*n* = 48 34 ZA, mean age 50.19 (±13.12), BML size 69.74 cm^3^ (±75.4); 14 placebo, mean age 53.6 (±7.1), BML size 46.99 cm^3^ (±76.8);	12 weeks	The addition of ZA to a regimen of suspended weight bearing and vitamin D has the potential to augment the healing capabilities of painful BMLs significantly by leading to a significant reduction in BML volume
Varenna et al., 2015 [37]	Double blind RCT	I	>50 years, knee OA, worsening of knee pain for at least 2 weeks, knee MRI scan showing large (>1 cm) BMLs	Neridronate 100 mg/8 mL ampoules or placebo, every third day starting on day 1 and ending on day 10	VAS, WOMAC, SF-36, WORMS, McGill	*n* = 68 34 neridronate, mean age 64.7 (±11.8), BMI 25.7 (±3.6); 34 placebo, mean age 67 (±7.3), BMI 25.5 (±3.9)	2 months	IV neridronate course showed a clinically relevant benefit in patients with acute painful OA, reducing the extent of bone marrow edema
Varenna et al., 2021 [38]	Double blind RCT	I	CRPS— International Association for the Study of Pain (IASP) diagnostic criteria	Neridronate 25 mg IM or matched placebo IM daily day for 16 consecutive days	VAS, SF-36, edema-score, McGill, adverse events	*n* = 78 41 neridronate, mean age 59.3 (±10.2), BMI 26.7 (±5.1); 37 placebo, mean age 59.7 (±10.5), BMI 26.6 (±4.4)	1 month	Clinically significant improvement in patients treated with neridronate compared with placebo
Varenna et al., 2022 [39]	Prospective cohort study	II	CRPS—International Association for the Study of Pain (IASP) diagnostic criteria	Patients treated with placebo during the double-blind phase of the study with IV neridronate at a dose of four 100 mg infusions each diluted in a 500 mL saline isotonic solution and infused over 2 h every third day	VAS, SF-36, edema-score, McGill, adverse events	*n* = 60 35 previous neridronate; 25 previous placebo now open label neridronate	12 months	Patients with acute CRPS-1 experienced a significant, clinically relevant, and persistent benefit following both IM and IV neridronate treatment regimens
Vasiliadis et al., 2021 [2]	Case series	IV	Bone marrow edema syndrome	IV 100 mL of ZA 5 mg/100 mL	VAS, adverse events	*n* = 54 54 ZA, mean age 52.7 (±9.7), BMI 28.9 (±3.8)	6 months	A single dose of intravenous ZA, when combined with partial weight-bearing for one month, yields favorable responses in both clinical and radiographic outcomes for patients with BMLs

ZA: zoledronic acid; BML: bone marrow lesion; VOLT01: methylprednisolone; CRPS: complex regional pain syndrome; BMI: body mass index; OA: osteoarthritis; BMD: bone marrow density; VAS: visual analog scale; WOMAC: Western Ontario and McMaster Universities Arthritis Index; KOOS: Knee Injury and Osteoarthritis Outcome Score; WORMS: Whole-Organ Magnetic Resonance Imaging Score; QoL: quality of life; PDI: pain disabilty index.

#### 3.1.1. Bisphosphonates in Bone Marrow Edema 

Seefried et al. published a triple-blind, placebo-controlled RCT on 48 patients showing that administration of a single-dose intravenous zoledronic acid (ZA) significantly reduced BML volume at 12 weeks compared to placebo (*p* = 0.009). Accordingly, visual analog scale (VAS) reduction was significantly greater in the ZA group (*p* = 0.042). Other PROMs did not reach statistical significance. The biochemical analysis revealed a statistically significant decrease in serum calcium levels in the ZA treatment group compared to placebo (*p* = 0.011). No statistically significant differences were observed between the ZA and placebo groups for the other biochemical measures including alkaline phosphatase, creatinine, C-reactive protein, and phosphate [36].

Vasiliadis et al. demonstrated a significant decrease in VAS at 6 months (7.25 ± 7.25 at baseline compared to 4.25 ± 1.84 at 6 months, *p* < 0.001) with a single-dose ZA in 54 patients affected by bone marrow edema syndrome of the knee in their case series. No major adverse events were reported [2].

Baier et al. performed a retrospective cohort study comparing monthly ibandronate to 5 days of iloprost in 20 patients affected by bone marrow edema of the knee and ankle. Iloprost is a synthetic analog of prostacyclin (PGI2) that mimics prostacyclin, a potent vasodilator produced in the vascular endothelium. At 12 months, both treatments improved VAS [6.4 (±1) to 1.1 (±0.9) in iloprost vs. 5.6 (±0.8) to 1.5 (±1.6) in ibandronate], WOMAC [53.6 (±11.5) to 12.1 (±11) in iloprost vs. 50.5 (±10.5) to 20.8 (±18.6) in ibandronate] and SF-36 scores [29 (±14.7) to 80.9 (±18.4) in iloprost vs. 24.3 (±12.8) to 80.9 (±23.4) in ibandronate]. Although iloprost acted more rapidly, showing statistically significant advantages at 3 months, no statistical inter-group difference was found at the final endpoint of 12 months [1].

In a retrospective cohort study, Müller et al. evaluated various antiresorptive medications including ibandronate, ZA, denosumab, and alendronate in 34 patients with symptomatic knee BMLs. None of the reported variables reached significant differences, probably due to the small sample size [34].

Bartl et al. performed a prospective cohort study of 50 patients with knee or ankle bone marrow edema syndrome (BMES). They found that 12 months of ibandronate significantly improved pain; VAS improved from 8.5 to 1.2 in the knee ibandronate group vs. 8.1 to 4 in the control groups. Larson knee scores improved from 54 to 89 and 51 to 70 respectively. BME Stage decreased from stage 2.9 to 0.8 in ibandronate at 6 months compared to Stage 2.8 to 2.2 in the diclofenac group [29].

#### 3.1.2. Bisphosphonates in Osteoarthritis

Looking at RCTs, Cai et al. performed a multicenter, double-blind RCT involving 223 OA patients with BMLs. The study revealed no significant difference in cartilage loss reduction, WOMAC, VAS, or BME after 2 years of yearly administration of ZA versus placebo [30]. Similarly, the same group previously performed a single-center, double-blind RCT in 117 OA patients and found that a ZA infusion did not lead to a significant higher incidence of adverse events compared with 5 mg ZA and 10 mg methylprednisolone (VOLT01). Lesion size at 6 months between ZA compared to VOLT01 and placebo showed no significant difference between the respective groups. However, VOLT01 showed statistically significant improvements in WOMAC score compared to both placebo and ZA, as well as statistically significant improvements in VAS compared with ZA [31].

Conversely, a single-center, double-blind RCT by Laslett et al. on 59 OA patients demonstrated that a single intravenous ZA infusion significantly reduced pain at 6 but not at 12 months, whereas the KOOS score trend showed no significant difference between groups. Similarly, BML size was significantly less than in the placebo group only at 6 months. Adverse events were more common in the ZA group (*p* = 0.001) [32].

Varenna et al. conducted a double-blind RCT of 68 OA patients that showed a significantly greater VAS decrease in patients receiving intravenous neridronate (*p* < 0.001) compared to placebo. Similarly, WOMAC (*p* = 0.001), McGill (*p* = 0.001), and WORMS (*p* = 0.002) showed also significant improvement vs. placebo, and only the overall SF-36 score showed no inter-group difference [37].

Ballal et al. carried out a retrospective cohort study on 290 matched individuals treated with and without bisphosphonates. Bisphosphonates did not clearly reduce BML size at 12 months compared to controls (difference in the mean change in total BML volume was 98.8 mm^3^, (95% CI: 156.6 to +354.2), *p* = 0.4). However, bisphosphonate users underwent a higher BML volume reduction rate (48%) compared to non-users (41%) [28].

#### 3.1.3. Bisphosphonates in Osteonecrosis of the Knee

Meier et al. performed a double-blind RCT on 30 patients with spontaneous osteonecrosis of the knee, which did not demonstrate significant benefits of ibandronate over anti-inflammatory medications in VAS, WOMAC, or IKDC-subjective scores up to 48 weeks. At 12 weeks, the biomarker of bone turnover, CTX, was significantly lower in the ibandronate group (*p* < 0.01), whereas other markers (PINP and ALP) presented similar concentrations (*p* = 0.06 and *p* = 0.71 respectively) [33].

Agarwala et al. conducted a prospective case series showing that a combination of a single intravenous ZA administration with weekly oral alendronate led to faster recovery in 16 patients with spontaneous osteonecrosis of the knee (SPONK). Pain evaluated by the VAS significantly improved at 16 weeks (*p* = 0.03), and the area of bone marrow involvement also significantly decreased at 16 weeks (*p* = 0.03) [27].

#### 3.1.4. Bisphosphonates in Transient Osteoporosis

Ringe et al. published a case series including 12 patients affected by localized transient osteoporosis and found ibandronate provided significant pain relief at 6 months compared to baseline VAS, although the increase in subchondral bone density did not reach statistical significance. No adverse events related to ibandronate were reported [35].

#### 3.1.5. Bisphosphonates in Complex Regional Pain Syndrome of the Knee

Transitioning to complex regional pain syndrome (CRPS), a double-blind RCT of 78 patients by Varenna et al. revealed that the neridronate group had significantly greater VAS reduction (*p* = 0.0003) and smaller BML (*p* = 0.03) at 1 month compared to placebo. SF-36 and McGill did not reach statistical significance. Adverse event incidence was not significantly different between groups [38].

After unblinding the groups, patients previously randomized to placebo received intravenous neridronate and both groups (previous IV neridronate and cross-over patients) were then evaluated up to 12 months. Each domain of SF-36 form reached statistically significant amelioration, except for the ‘General Health’ domain. No difference between the two treatment arms were observed [39].

### 3.2. Meta-Analysis

**Figure 3:**
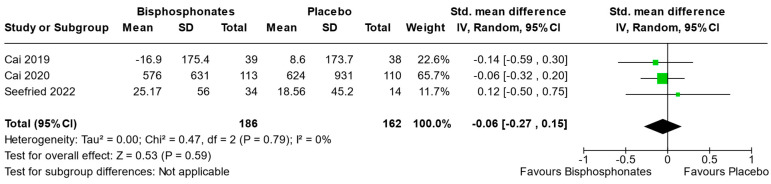
Forest plot of the individual studies and pooled mean difference for BML size, with a 95% confidence interval. The size of the squares shows the weight of the study. The random-effects model was used due to the inherent heterogeneity in the design and methodology of the included studies.

Three studies (Cai et al., 2019 [31], Cai et al., 2020 [30], and Seefried et al., 2022 [36]) reported sufficient data to perform meta-analysis on effects of bisphosphonates on BML size. The analysis focused on BML size at the end of the follow-up period (6, 24, and 1 month, respectively), using a comparison between two intervention groups. The analysis demonstrated that bisphosphonates were not able to significantly reduce BML size compared to placebo (Figure 3).

**Figure 4:**
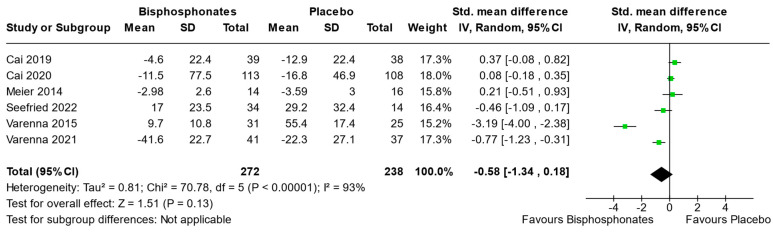
Forest plot of the individual studies and pooled mean difference for Visual Analogue Scale (VAS) for pain improvement, with a 95% confidence interval. The size of the squares shows the weight of the study. The random-effects model was used due to the inherent heterogeneity in the design and methodology of the included studies.

Six studies (Cai et al., 2019 [31], Cai et al., 2020 [30], Meier et al., 2014 [33], Seefried et al., 2022 [36], Varenna et al., 2015 [37], and Varenna et al., 2021 [38]) reported sufficient data to perform meta-analysis on VAS for pain. The analysis focused on VAS at the final follow-up evaluation (6, 24, 3, 1, 1 and 1 month respectively), comparing the two intervention groups. The results showed a slight no significant advantage of bisphosphonates compared to placebo (Std. mean difference [95% CI] −0.58 [−1.34, 0.18]) (Figure 4).

**Figure 5:**
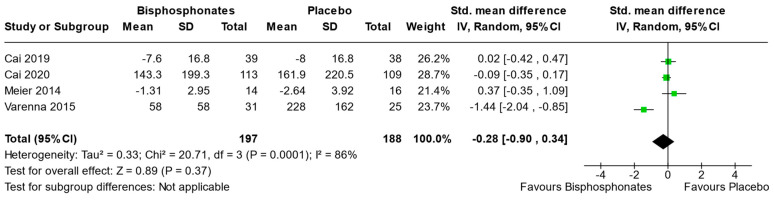
Forest plot of the individual studies and pooled mean difference for WOMAC, with a 95% confidence interval. The size of the squares shows the weight of the study. The random-effects model was used due to the inherent heterogeneity in the design and methodology of the included studies.

Four studies (Cai et al., 2019 [31], Cai et al., 2020 [30], Meier et al., 2014 [33], and Varenna et al., 2015 [37]) reported sufficient data to perform meta-analysis on WOMAC. The analysis focused on WOMAC at the final follow-up evaluation (6, 24, 3, and 1 month, respectively). The results indicated a non-significant advantage of bisphosphonates compared to placebo (Std. mean difference [95% CI] −0.28 [−0.9, 0.34]) (Figure 5).

**Figure 6:**
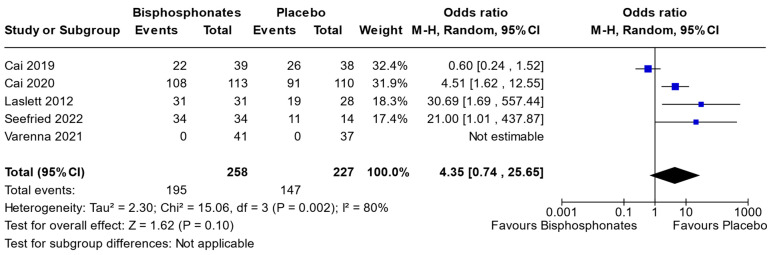
Forest plot of the individual studies for adverse events, with a 95% confidence interval. The size of the squares shows the weight of the study. The random-effects model was used due to the inherent heterogeneity in the design and methodology of the included studies.

In the context of this meta-analysis, we scrutinized data from five distinct studies (Cai et al., 2019 [31], Cai et al., 2020 [30], Laslett et al., 2012 [32], Seefried et al., 2022 [36], and Varenna et al. 2021 [38]) that provided sufficient information for the evaluation of adverse events (AEs). The primary objective was to discern any AE linked to the administration of bisphosphonates compared to placebo. Our findings demonstrated no statistically significant association between AEs and the use of bisphosphonates in contrast to placebo. Notably, no subgroup analyses or supplementary filters were applied during this analysis (odds ratio [95% CI] 4.35 [0.74, 25.65]) (Figure 6). 

The most commonly observed side effects were flu-like symptoms and arthralgia; none of the patients reported atypical fracture of the jaw.

## 4. Discussion

The main finding of the present meta-analysis is that bisphosphonates are not able to significantly reduce BML size compared to placebo, and even in terms of clinical response, evaluated by pain and subjective scores, no significant benefit emerged over the control group. Accordingly, a non-significant higher rate of AEs was documented after bisphosphonate administration. The heterogeneity tests reveal some important insights about the variability across the included studies. For the meta-analysis on bone marrow edema size, there was no significant heterogeneity (I² = 0%), indicating consistent effects across studies. However, substantial heterogeneity was observed in the meta-analyses for clinical outcomes like VAS pain scores (I² = 93%), WOMAC scores (I² = 86%), and other subjective measures (I² = 80%). The high degree of heterogeneity for these patient-reported outcomes suggests considerable variability in the effects reported across studies, which could be attributable to differences in patient populations, study designs, or other clinical factors. While bisphosphonates did not demonstrate significant benefits over placebo in reducing BML size or improving clinical outcomes, the presence of substantial heterogeneity, particularly for patient-reported measures, warrants cautious interpretation.

Bisphosphonates are anti-resorptive drugs borrowed from the treatment regimen for osteoporosis [40,41,42], but they also demonstrated a particular “reconstructive” effect on the periarticular bone [43]. Given the strong correlation between articular cartilage and subchondral bone [44,45], the first application was therefore the treatment of the subchondral pathology in knee OA. Encouraging preclinical studies [46,47,48] were followed by human application [23,43,49] which, however, did not show such exciting results. One of the major reasons for this discordance lies in the nature of OA itself, which entails a wide spectrum of alterations, and therefore the evaluation of response is likely to be influenced by the concomitant cartilage and synovial tissue damage [50,51]. However, BMLs are not only observed in cases of OA, but they are also a common MRI finding in a variety of pathologies which are sometimes difficult to distinguish. In fact, the previously called ‘bone marrow edema’ has nothing to do with fluid extravasation in the subchondral bone, but rather englobes peculiar ultrastructural and histological changes [52]. The modern concept of subchondral bone alterations includes a continuum of pathologies where post-traumatic and OA-related BMLs are at the extremities [12]: transient osteoporosis, regional migratory osteoporosis, complex regional pain syndrome (CRPS), and algodystrophy are considered reversible conditions, [53] whereas spontaneous osteonecrosis of the knee, avascular necrosis, and insufficiency fractures are mostly considered irreversible [22]. Despite the wide range of diseases grouped under the definition of “bone marrow lesions”, the pathological changes seen in each of the above are sustained by similar mechanisms, which are targeted by bisphosphonates. Indeed, from a qualitative evaluation of the articles retrieved, the use of bisphosphonates offers significant amelioration in terms of clinical response for the treatment of isolated BMLs. Accordingly, the potential of these drugs is also evident when BMLs are associated with OA. In fact, two [30,31] out of three RCTs analyzing the reduction in BML size are on knee OA and documented a “borderline” significant reduction in BML size when bisphosphonates were used, hence raising hopes on the potential benefits of these drugs in OA-associated BMLs. Moreover, although our meta-analysis yielded no statistically significant clinical benefits from bisphosphonate use, none of the studies reported information on the Minimal Clinically Important Difference (MCID) [54], which appears to be a much more prominent approach to critically present the effects of an intervention rather than a mere statistical test. Furthermore, when focusing on the type of bisphosphonate used, seven out of 15 studies reviewed here adopted ZA and, more interestingly, the only results close to statistical significance in terms of BML size were documented in studies employing ZA [30,31,36], thus supporting the thought of its potential superiority over other drugs of the same class. Nonetheless, we feel that the peculiar characteristics and the potential harms and benefits of specific bisphosphonates for the treatment of BMLs still needs to be clearly elucidated. Indeed, although the adverse events related to bisphosphonates are not truly a major concern, since they are well-known following their extensive use in the treatment of osteoporotic patients, orthopedic surgeons are usually more cautious when prescribing molecules whose first indication is a bone metabolism disease such osteoporosis: this is due to the fact that osteoporosis is a systemic disease, whereas BMLs are always localized and therefore, from the perspective of a surgeon, a “systemic” drug may fail to sufficiently reach the injured site, thereby exposing the patient to the risk of undesired adverse events of such drugs. This demands further investigations to identify the right dosages and timing of bisphosphonate application in BMLs in order to maximize their efficacy. To date, some significant results have been achieved: in fact, the trials on neridronate published by Varenna et al. [37,38,39] reported such brilliant results for the treatment of CRPS that this drug has received approval, in some countries, as the only labeled drug for the treatment of CRPS. 

The present systematic review and meta-analysis is not free from limitations. Firstly, we analyzed studies that evaluated the effectiveness of bisphosphonates in the different pathologies of BML spectrum. However, the differential diagnosis among the above-mentioned diseases is often difficult to apply in clinical practice due to the lack of standardized diagnostic criteria for each of them. Hence, we preferred to evaluate the impact of bisphosphonates in the treatment of BMLs irrespective of any clinical classification and also because to our knowledge, there is no evidence that any clinical entity has higher or lower chances of response following its administration. One further burden is represented by the different timing of administration from symptom onset, which may itself impact the results. One further limitation is given by publication bias, as studies with significant or positive results are more likely to be published and included, which may overestimate the true effect size. Going into the specifics of the single studies included, the overall risk of bias for the majority of the included articles is still far from high-level evidence. Furthermore, the different molecules used, the variable dosage and diverse ways of administration, represent adding confounding variables preventing us from standardizing the available results. Lastly, we included multiple studies by the same authors that adopted similar inclusion criteria, which could suggest that the same patient population was utilized for multiple studies.

## 5. Conclusions

Bisphosphonates show neither significant benefits nor significant adverse events when compared to placebo in the treatment of BMLs of the knee. 

## Figures and Tables

**Table 2:** ROBINS-I tool for the risk of bias assessment of the included studies that were not RCTs.

Publication	Baseline Confounding	Selection of Participants	Classification of Intervention	Deviation from Intended Intervention	Missing Data	Measurement of Outcomes	Selection of Reported Results	Overall Risk of Bias
Agarwala et al. (2020) [27]	Low	Low	Low	Low	No information	Low	Low	Low
Baier et al. (2013) [1]	Low	Moderate	Low	Low	No information	Low	Moderate	Moderate
Ballal et al. (2020) [28]	Moderate	Serious	Low	Low	Low	Moderate	Low	Serious
Bartl et al. (2012) [29]	Moderate	Low	Low	Low	No information	Moderate	Low	Moderate
Müller et al. (2020) [34]	Moderate	Moderate	Moderate	Low	Low	Low	Moderate	Moderate
Ringe et al. (2005) [35]	Low	Low	Low	Low	No information	Low	Low	Low
Varenna et al. (2022) [39]	Low	Low	Low	Low	Moderate	Low	Moderate	Moderate
Vasiliadis et al. (2021) [2]	Low	Low	Low	Low	No information	Low	Low	Low
